# Common Warts and Their Treatment

**Published:** 1897-12

**Authors:** M. B. Hutchins

**Affiliations:** Clinical Lecturer on Skin Diseases and Syphilis, Atlanta Medical College, Atlanta, Ga.


### COMMON WARTS AND THEIR TREATMENT.

BY M. B. HUTCHINS, M.D.,

Clinical Lecturer on Skin Diseases and Syphilis, Atlanta Medical College, Atlanta, Ga.

"Warts of various kinds, so tedious of treatment by the old methods, save that of excision, with or without cauterization, usually dissolve into froth under the action of electrolysis, being more easily destroyed than the average mole." This statement was made in a paper which I read before The Medical Association of Georgia in April, 1896, and published in *The Southern Medical Record* for May, 1896, and in *The Transactions* for that year.

While this claim for electrolysis is true in part, exceptions have been of sufficient frequency to excuse a somewhat fuller discussion of the subject.

Warts which are rather soft, showing but little hypertrophy of epidermis, and chiefly composed of connective tissue, do yield very readily to the negative galvanic current applied through the needle, even if the current is not over one-half to one milliampere. Such warts readily "dissolve" under the current, and the chances are wholly in favor of their permanent cure.

When, however, we come to treat the hard, dry, horny kind of wart, we find quite a different pathological condition, and obtain less favorable results. We may have much frothing around the needle—if it reaches the connective tissue parts of the growth—

and the wart may "slough out," a dry eschar, later, but we are often confounded by the regrowth of the lesion. It would seem that these horny, dry warts possessed downward projections of epithelial cells; a specific germ, or that we failed to reach, with our weak current, the vital point.

Analogy may help to explain the result. A cut-off finger does not regrow. A positive destruction of the true skin leaves a scar. These organs are of mesoblastic origin. The hair and nails are constantly cut and constantly regrow. They are epithelial structures, from the embryonic epiblast.

A mole, connective tissue growth, does not regrow from any portion which may be left behind. A wart of the largely epithelial kind will regrow after partial destruction by any agent.

Thorough destruction of this kind of wart ends it. We can get this destruction by a sufficiently prolonged treatment with weak electrolysis in a sufficient number and depth of punctures or transfixions, or we can obtain the desired result with a current so strong that its action is practically that of a chemical caustic.

If the wart be very dry and hard, electrolysis is slower and more painful than some other method.

If the dry top of the wart be first cut away, any sufficiently strong caustic will destroy it. If the patient objects to the cutting, caustic potash, which readily attacks horny tissue, will thoroughly destroy the lesion, if properly used. Salicylic acid may not be expected to affect the papillary part of the wart, as its action is upon the horny cells almost exclusively.

The common hard wart is peculiar—in that it goes away when its time comes under all kinds of conjuring and hoodooing, from the lick of a puppy to rubbing a dying person's face.

But when it is not ready to pass away, more scientific and active remedies must be resorted to.

*311–312 Fitten Building.*

---

"They tell me, Professor, that you have mastered all of the modern tongues."

Professor—All but two—my wife's and her mother's.—*Ex.*

